# Dry Generation of CeO_2_ Nanoparticles and Deposition onto a Co-Culture of A549 and THP-1 Cells in Air-Liquid Interface—Dosimetry Considerations and Comparison to Submerged Exposure

**DOI:** 10.3390/nano10040618

**Published:** 2020-03-27

**Authors:** Francesca Cappellini, Sebastiano Di Bucchianico, Venkatanaidu Karri, Siiri Latvala, Maria Malmlöf, Maria Kippler, Karine Elihn, Jonas Hedberg, Inger Odnevall Wallinder, Per Gerde, Hanna L. Karlsson

**Affiliations:** 1 Institute of Environmental Medicine, Karolinska Institutet, Stockholm, 17177 Sweden; 2 Comprehensive Molecular Analytics, Helmholtz Zentrum München, 81379 München, Germany; 3 Department of Environmental Science, Stockholm University, Stockholm11418, Sweden; 4 Inhalation Sciences, Hälsovägen 7-9, 141 57 Huddinge, Sweden; 5 KTH Royal Institute of Technology, Department of Chemistry, Division of Surface and Corrosion Science, 114 28 Stockholm, Sweden

**Keywords:** nanotoxicology, air-liquid interface, PreciseInhale, dosimetry, inflammation, ceria

## Abstract

Relevant in vitro assays that can simulate exposure to nanoparticles (NPs) via inhalation are urgently needed. Presently, the most common method employed is to expose lung cells under submerged conditions, but the cellular responses to NPs under such conditions might differ from those observed at the more physiological air-liquid interface (ALI). The aim of this study was to investigate the cytotoxic and inflammatory potential of CeO_2_ NPs (NM-212) in a co-culture of A549 lung epithelial cells and differentiated THP-1 cells in both ALI and submerged conditions. Cellular dose was examined quantitatively using inductively coupled plasma mass spectrometry (ICP-MS). The role of serum and LPS-priming for IL-1β release was further tested in THP-1 cells in submerged exposure. An aerosol of CeO_2_ NPs was generated by using the PreciseInhale^®^ system, and NPs were deposited on the co-culture using Xpose*ALI*^®^. No or minor cytotoxicity and no increased release of inflammatory cytokines (IL-1β, IL-6, TNFα, MCP-1) were observed after exposure of the co-culture in ALI (max 5 µg/cm^2^) or submerged (max 22 µg/cm^2^) conditions. In contrast, CeO_2_ NPs cause clear IL-1β release in monocultures of macrophage-like THP-1, independent of the presence of serum and LPS-priming. This study demonstrates a useful approach for comparing effects at various in-vitro conditions.

## 1. Introduction

The production and use of different nanoparticles (NPs) is steadily increasing in society, leading to an urgent need for reliable assessment of their toxicological properties. Exposure via inhalation is considered the most common exposure route in humans, particularly in occupational settings, and therefore methods simulating lung exposures are needed [1]. Most in vitro studies applied today use lung cells exposed to particles in a liquid suspension, i.e., submerged exposures. Such exposure leads to interactions between the medium and the NPs, often resulting in agglomeration, corona formation, dissolution etc. In addition, the preparation of the NP suspension often involves sonication and the method used will affect the NP properties [2], as will the use of serum [3,4]. Furthermore, under submerged conditions, NPs may be attached to the wall of the cell exposure plate, and can indeed also remain in the liquid, and thus never reach the cells [1,2,5,6]. A more realistic scenario for assessing lung cell toxicity is to use direct exposure of the cells in the air-liquid interface (ALI). In this case, the cells are cultured on inserts with no cell media covering the cells, which enables exposure of the cells to an aerosol of particles. Several ALI systems have been described in the literature using different methods for aerosol generation and types of cell exposure units [7,8,9]. There are, however, many challenges with ALI systems, including difficulties generating dry nano-aerosols from powders and depositing these on the cells.

Cerium dioxide nanoparticles (CeO_2_ NPs) are important in various applications, such as in diesel fuel as catalysts able to increase the fuel combustion efficiency and to reduce the emissions of soot and other particles, in polishing of crystalline silicon wafers, and as UV-absorbents [10,11]. The toxicological profile of CeO_2_ NPs in the literature shows divergent results, with several studies reporting anti-oxidant and protective effects [12,13], whereas others report inflammatory and toxic effects. In animal studies, inflammatory effects have been observed following intratracheal instillation [14] and inhalation [15], and rats appear to be more sensitive than mice [16]. There is, however, a strong need to replace animal studies with cellular studies, and thus an important question concerns which in vitro models that are the most relevant to use Exposure in ALI appears to be more realistic, but there are few studies that directly compare effects observed in submerged conditions and ALI. In a study on different NPs (TiO_2_ and CeO_2_), Loret et al. (2016) concluded that significant pro-inflammatory responses were observed at lower deposited doses in ALI compared to the submerged exposure [17]. On the other hand, another study on amorphous silica NPs showed that the submerged exposure led to stronger effects observed at lower cellular doses [18].

This study aimed to test the cytotoxic and inflammatory potential of a CeO_2_ reference nanomaterial (NM-212) in a co-culture of A549 and THP-1 cells. In particular, the study aimed to compare submerged and ALI conditions with a proper dose comparison (cell dose assessed quantitatively). The role of serum and priming for IL-1β release was further tested in THP-1 cells in submerged exposure. We hypothesized that both the exposure system (ALI vs. submerged) and the use of serum and priming may affect the ability to detect an inflammatory effect of CeO_2_ NPs.

## 2. Materials and Methods

### 2.1. CeO_2_ NPs Information and Physico-Chemical Properties

Well characterized uncoated cerium(IV) oxide (CeO_2_) NPs were acquired from the repository of the European Commission Joint Research Centre, Institute for Health and Consumer Protection (JRC-IHCP; Ispra, Italy). The physico-chemical characteristics of the as-received CeO_2_ NPs (NM-212) are thoroughly described in [19]. In short, the as-received NPs (dry) are highly agglomerated, with irregular (polyhedral morphology) and non-homogeneous particle sizes ranging from below 10 nm to >100 nm (based on transmission electron microscopy measurments). The particles have a mean primary diameter of 28.4 ± 10.4 nm (based on Feret’s diameter measurements by means of scanning electron microscope and a specific surface area (BET) of 27.2 ± 0.9 m^2^/g. Compositional analyses of the outermost surface (top 10 nm) show Ce(IV) to be the dominating oxidation state (>95%), although some contribution from Ce(III) cannot be excluded as influencing the catalytic properties of the particles.

### 2.2. Cell Culture and Reagents

Cell culture medium (RPMI 1640, Lot# 1720776), phosphate buffered saline (PBS), fetal bovine serum (FBS, Gibco^®^, Lot# 07F2235K), Penicillin/Streptomycin antibiotics mixture (Gibco^®^), and L-glutamine (Gibco^®^) were purchased from Life Technologies (Stockholm, Sweden). Alamar blue (Lot #GF218932) was purchased from Thermo scientific. The LDH (lactate dehydrogenase) activity kit (MAK066, Lot #B2C300726V) and Phorbol 12-myristate 13-acetate (PMA) were purchased from Sigma-Aldrich (Stockholm, Sweden, and the Luminex assay (Bio-Plex^®^ Multiplex System, Lot #64020782) from Bio-Rad (Solna, Sweden). HNO_3_ (67–69%, trace-metal grade, Lot #1113070) was bought from Fisher Chemical (Fischer Scientific, Loughborough, UK Limited).

A549 cells (human type II alveolar epithelial cell line) were obtained from NANoREG partners (Bundesanstalt Fuer Arbeitsschutz und Arbeitsmedizin, BAuA, Berlin, Germany) and THP-1 cells (peripheral blood monocyte cell line) were obtained from the American Type Culture Collection (Gaithersburg, USA).

### 2.3. Preparation of CeO_2_ Dispersions

The NP suspensions for the submerged treatments in the transwells were prepared according to the protocol used within the project NANoREG. In short, the NPs were suspended in 0.05% bovine serum albumin (BSA) at a concentration of 2.56 mg/mL and then sonicated for 15 min and 30 s using an MSE Soniprep 150 equipped with an exponential microprobe (Microtip type 38121-114A) at a 22 micron amplitude in a continuous mode. This setup resulted in an average power of 7.56 (±0.01) W and a sample specific energy of 7056 J, according to calibrations performed in the frame of the NANoREG project (developed by K. Jensen and co-workers, The National Research Centre for the Working Environment, Copenhagen, Denmark). During sonication, the samples were cooled in icy water to prevent excessive heating. For the submerged exposures performed on monocultures of THP-1 cells, the NPs were weighed and a medium (with or without serum) was added to a final concentration of 1 mg/mL. The suspensions were then vortexed and sonicated in bath for 10 min twice. The suspensions were diluted to final concentrations and immediately used in the cell experiments.

### 2.4. Size Characterization in Cell Medium

Photon cross correlation spectroscopy (PCCS) was used to investigate size distribution and sedimentation of the NPs, prepared according to the described protocol and diluted to 20 µg/mL in a cell culture medium (with or without serum). The samples were prepared in disposable single sealed cuvettes, LOTG17501P (Eppendorf AB), and measured in triplicate immediately after preparation, as well as after 24 h using the NANOPHOX 90-250 V (Sympatec, Clausthal Germany) instrument. The software Windox (Clausthal, v.5) was used to obtain the size distribution data for each measurement. Measurements of standard latex particles (100 nm) were performed to ensure accurate measurements of particle size.

### 2.5. Preparations of Co-Cultures of A549 and THP-1 Cells

The cells were cultured at 37°C in a humidified 5% CO_2_ atmosphere in an RPMI 1640 cell culture medium (supplemented with 10% (v/v) FBS, 1% (v/v) L-glutamine, and 1% (v/v) Penicillin/Streptomycin mixture). THP-1 cell concentrations were maintained between 2–8 × 10^5^ cells/mL. A549 cells were seeded at a concentration of 30,000 cells/cm^2^ in a 500 µL medium on the apical side of each transwell (Falcon^®^ Cell Culture Inserts, Corning^®^, for 12 well plates, Cat #353494, high-density, 0.4 µm pore size, transwell diameter 0.9 cm^2^, supplied by VWR, Stockholm, Sweden). The volume of the cell culture medium in the basal chamber was 1.2 mL. The cells were cultured for 72 h, after which the medium was changed both on the apical and basal sides of the transwells (500 and 900 µL, respectively). Thereafter, the cells were cultured for another 24 h. Meanwhile, THP-1 cells in cell culture flasks (75 cm^2^) were differentiated with 300 ng/mL PMA for 24 h. The THP-1 cells were then trypsinized, and 42,000 cells/well in a 500 µL medium were seeded on the A549 cell cultures on the apical side of the transwells. This yielded an A549: THP-1 ratio of approximately 10:1. The medium in the basal chamber was removed and 850 µL of fresh medium was added. For the ALI transwells, the medium was removed from the apical side 4 h after adding the THP-1 cells, and the cells were allowed to adapt to the ALI conditions for 20 h before the ALI treatment. For the submerged transwells, the medium was kept on both sides of the transwells before the treatment.

### 2.6. Submerged Exposures in Inserts and Plates

The submerged treatments of co-cultures in inserts were performed by removing the medium from the apical side and directly adding the NP suspensions on the cells (500 µL). All submerged treatments were performed with 500 µL of NP suspension on the apical side and 850 µL medium on the basal side. The applied doses of CeO_2_ in the submerged exposures were 2, 10, 20, 30, and 40 μg/cm^2^. Positive controls were treatments with 0.1% triton-x and 100 ng/mL lipopolysaccharide (LPS, from Sigma). Incubator and sham controls for both ALI and submerged treatments were used as negative controls. For exposure of THP-1 cells in plates, 40,000 cells were seeded in a 96-well plate in 100 μL, and 5 ng/mL PMA was added for differentiation for 24 h. In the case of priming, 100 ng/mL of LPS was added for 4 h. The cells were then exposed to 2, 10, 20, 30, and 40 μg/cm^2^ (with or without serum) for 24 h.

### 2.7. Aerosol Generation and Deposition in ALI

**Figure 1:**
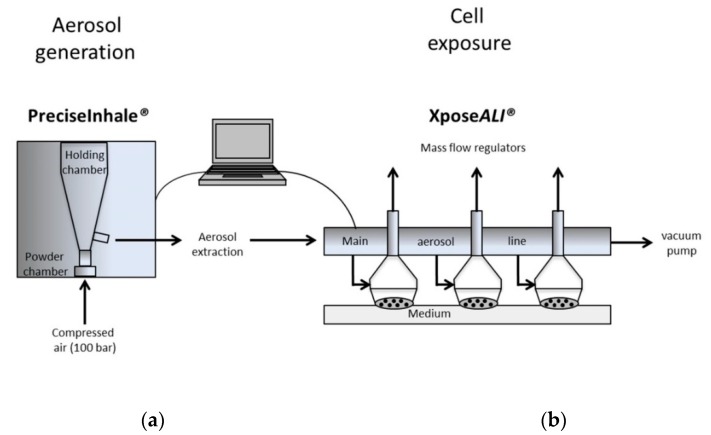
Exposure of cells to an aerosol of cerium dioxide nanoparticles (CeO_2_ NPs) generated from dry powders in the PreciseInhale system (**a**) in combination with the Xpose*ALI* cell exposure unit (**b**). A small amount of powder (typically 2 mg) is loaded into the powder chamber. An aerosol is generated using rapid decompression of powder agglomerates, and the delivered mass can be estimated by a light dispersion instrument. Deposition on cells (5 mL/min flow rate) is analyzed offline using inductively coupled plasma mass spectrometry (ICP-MS).

An aerosol was generated from dry CeO_2_ powder using the PreciseInhale^®^ system (Figure 1). A small amount of powder (typically 2 mg) was loaded into the powder chamber, and an aerosol was generated in the holding chamber using compressed air (100 bar), causing rapid de-agglomeration of the powder agglomerates. The aerosol was extracted out of the holding chamber and passed via a light dispersion instrument (Casella) to estimate the aerosol concentration. Thereafter, the aerosol was pulled through the main aerosol line of the cell exposure unit (Xpose*ALI*) with a by-pass flow rate of approx. 60 mL/min, controlled by a vacuum pump. The aerosol was delivered to the three exposure hoods via separate inlet tubes from the main aerosol line. The aerosol flow rate over the cells in the exposure unit was controlled by mass flow regulators (5 mL/min each). The excess of particles not deposited on the cell surface was caught on 6 mm end filters at the outlet of the exposure hoods. The exposure time in Xpose*ALI* varied between approx. 5–20 min, depending on the desired dose. After the exposure, the cells were transferred to a cell incubator to allow for further exposure in the ALI state for approx. 24 h before toxicity analysis. Both incubator controls (Ctrl) and clean air exposure (Sham) were used.

### 2.8. Scanning Electron Microscopy (SEM) and Energy Dispersive Spectroscopy (EDS)

To assess the deposition pattern of CeO_2_ NPs (generated via the PreciseInhale aerosol generator), collagen-coated cover slips were placed in the transwell inserts as cell surface surrogates. Inserted into the Xpose*ALI* module, a particle deposition procedure was performed using the same settings as for cell exposures. Scanning electron microscopy imaging was then performed using a FEI XL30 instrument equipped with an Oxford EDS application with INCA software. Compositional analysis was performed to confirm the Ce content of deposited particles. Before imaging, a thin gold layer (6 nm) was deposited on the slips to ensure proper conduction.

### 2.9. Quantification of Deposition/Cellular Dose Using Inductively Coupled Plasma Mass Spectrometry (ICP-MS)

Two transwell inserts from each exposure concentration both from the ALI and the submerged treatments were used for quantifying the NP deposition/cellular dose. The cellular dose is defined as NPs in or on the cells (remaining on the cell after removal of the medium). The chemical analysis of deposited CeO_2_ was performed with inductively coupled plasma mass spectrometry (ICP-MS; iCAP Q; Thermo Scientific, Waltham, MA, USA). Calibration was performed with standard solutions containing 0.1, 0.5, 1, 5, 10, 50, 100, and 500 µg/L of cerium in 5% HNO_3_ (TraceCERT^®^, Sigma Aldrich, prepared using high purity CeO_2_). Recovery of cerium (92% ± 8.7%) was tested prior to the analysis by adding 10 µg of CeO_2_ on transwell membranes followed by the same treatment and analysis procedure as for the samples. Indium (In) and rhodium (Rh) were used as internal standards at a final concentration of 5 µg/L in all standards and samples. The transwell membranes were cut out into a Falcon^®^ tube containing 1 mL of MilliQ water (18.2 MΩ cm). Thereafter, the membrane and storage solution was transferred into a quartz tube and digested with 2 mL of concentrated nitric acid (Scharlau Trace Analysis Grade, Scharlab, Sentmenat, Spain) and 3 mL MilliQ water for 30 min (250 °C and a pressure of 160 bar), using a Milestone ultraCLAVE II microwave digestion system (EMLS, Leutkirch, Germany). The digested samples were diluted with MilliQ water in order to reach a 5% HNO_3_ concentration. For the ICP-MS analysis, triplicate readings of each sample (RSD < 20%) were recorded with the kinetic energy discrimination (KED) mode to avoid interferences with any polyatomic molecular species. Blank and a cerium standard (5 µg/L) were run as quality control samples after every 10 samples. Internal standard recovery (In and Rh) levels were between 90% and 110%. Results are based on the concentrations measured for one of the most abundant cerium isotopes (Ce^142^), which showed the highest stability in the blank samples. The limit of detection (LOD) and limit of quantification (LOQ), calculated as 3 and 10 times the standard deviation (SD) of the matrix-matched control samples, were 0.01 and 0.03 µg/L, respectively. All cerium samples were blank-corrected by subtracting the average value of three blank samples from the measured concentration of each sample.

### 2.10. Analysis of Cytotoxicity and Inflammatory Potential in Co-Cultures

After the ALI exposure, the cells were exposed for additionally 24 h in 12 well plates with 850 µL of medium on the basal side. Thereafter, one transwell from each treatment condition was used for an Alamar blue cytotoxicity test. These transwells were transferred into a 6 well-plate and 400 µL of 10% Alamar blue solution in cell medium were added on the apical side, after which they were incubated for 3 h. Then the solution was mixed gently by pipetting, and 100 µL was transferred into a 96-well plate in triplicate wells for each transwell. Absorbance was recorded at 570 nm. Supernatants from each transwell were collected for LDH assay and cytokine release analysis. For the submerged samples, medium was collected both from the apical and the basal side of the transwell and centrifuged for 30 min at 13,000 rpm (11,600 g) at 4 °C. For the ALI samples, medium was collected from the basal side. For the LDH assay, 50 µL of the supernatant was used to quantify LDH leakage from the cells (quantified by analyzing the conversion of NAD+ to NADH in the presence of lactate) following the supplier’s instructions. The cytokine release analysis was performed by diluting 50 µL of supernatant 1:1 in medium. The assay was performed to test IL-1β, IL-6, IL-8, TNF-α, and MCP-1 production, following the supplier’s (Bio-Rad) instructions.

### 2.11. Analysis of Cytotoxicity and IL-1β Release in THP-1 Cells (in Plates)

After exposure, the medium was removed, briefly centrifuged, and the supernatant was frozen for IL-1β analysis. The viability of the cells was tested by using Alamar blue assay. IL-1β release was analyzed by using Human IL-1 beta/IL-1F2 DuoSet ELISA kit (R&D systems), according to the instructions from the manufacturer. The light absorbance was measured at 540 nm in a microplate reader (Tecan, Infinite F 200, Austira GmbH, Software: Magellan 7.2), and a linear standard curve was generated and used to determine the IL-1β concentration.

### 2.12. Statistical Analysis

The statistical analyses (one-way ANOVA) were performed in Prism5 Graphpad and the significance level was chosen at *p* < 0.05.

## 3. Results

### 3.1. Aerosol Generation and CeO_2_ Deposition in ALI and Submerge

**Figure 2:**
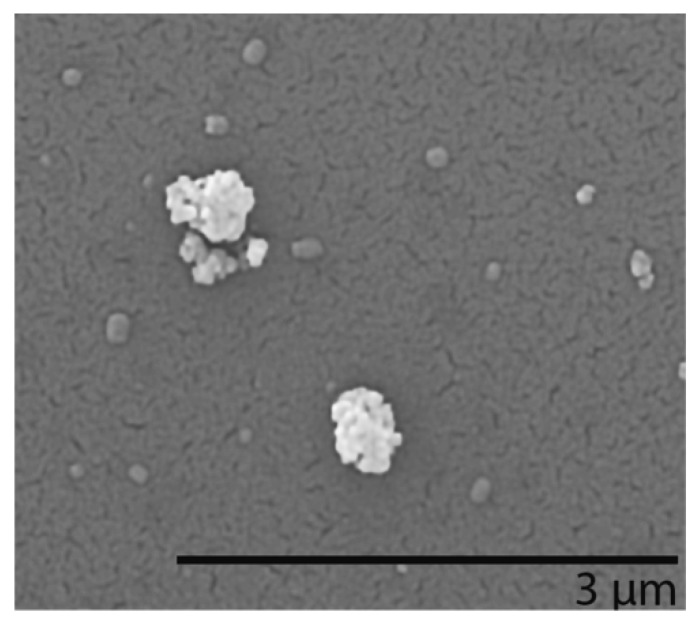
Scanning electron microscopy (SEM) images of CeO_2_ NPs aerosolized in the PreciseInhale system and deposited on collagen-coated cover slips put in transwells in the Xpose*ALI* unit.

By using the PreciseInhale in combination with Xpose*ALI*, shown in Figure 1, a dry aerosol could be generated and deposited on cells. First, the deposition of agglomerated CeO_2·_NPs on collagen-coated coverslips put in inserts (without cells) in the cell exposure unit was confirmed using light microscopy (data not shown), as well as the SEM/EDS analysis. The CeO_2_ NPs observed in the SEM analysis were mainly agglomerated, typically sized between 200 and 800 nm (see Figure 2). The deposition for the ALI exposures were approximately 0.5, 1, 2, and 5 μg/cm^2^, as quantified using ICP-MS (Table 1). In the submerged exposures (using probe sonication), the deposited amounts of CeO_2_ were 1, 5, 9, 15, and 22 μg/cm^2^, which was approximately 50% of the added amounts (2, 10, 20, 30, and 40 μg/cm^2^, Table 1).

### 3.2. CeO_2_ Characterization in Cell Medium

The mean particle size and extent of sedimentation of the CeO_2_ NPs (primary size 28 ± 10 nm) were investigated for the different investigated media and dispersion methods, Table 2. Preparation of a stock suspension in MilliQ and albumin using probe sonication followed by dilution in serum containing media showed a stable suspension of agglomerates approximately sized 240 nm, both at 0 and 24 h. Sonication in a water bath led to the formation of larger-sized agglomerates, typically at 800 nm for serum containing medium, and at several micrometers at serum-free conditions. These large agglomerates sedimented to a high degree with time, as observed from the low scattered light intensity observed at 24 h. These findings are in line with the deposited mass of CeO_2_ NPs determined by means of ICP-MS, Table 1.

### 3.3. Cytotoxicity

**Figure 3:**
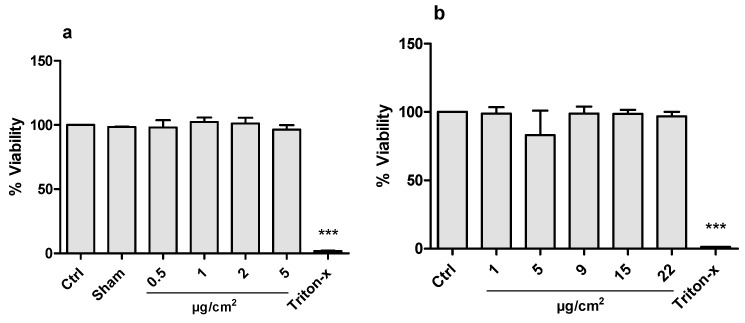
Cell viability analyzed by Alamar blue assay in co-cultured A549 and THP-1 cells exposed to CeO_2_ NPs for 24 h in air-liquid interface (ALI) (**a**) or in submerged (**b**) cell culture. Triton-x (0.1%) was used as positive control. Data is presented as % of the control value (Ctrl), and bars show mean ± SD. For ALI, clean air exposure (Sham) was used as additional control. Significant results as compared to the control are marked with asterisks (*** for *p* < 0.001).

**Figure 4:**
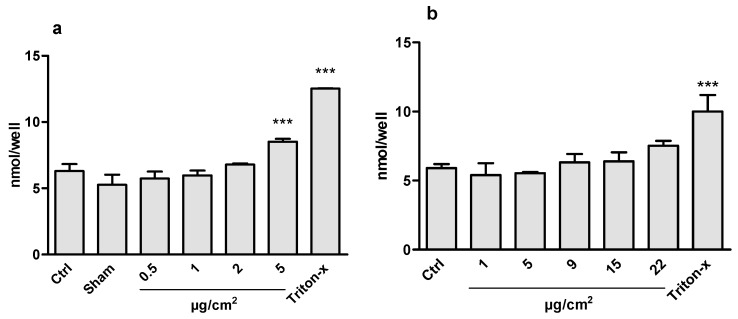
Cell viability analyzed by LDH assay in co-cultured A549 and THP-1 cells exposed to CeO_2_ NPs for 24 h in air-liquid interface (ALI) (**a**) or in submerged (**b**) cell culture. Triton-x (0.1%) was used as positive control. Data is presented as the amount of NADH (nmol) generated by the supernatant at a given time, and is proportional to the LDH activity. The bars represent mean ± SD. Significant results, as compared to the Sham (clean air) for ALI and control (Ctrl) for submerged, are marked with asterisks (*** for *p* < 0.001).

Exposure of the co-cultures to CeO_2_ NPs in ALI at concentrations of 0.5, 1, 2, and 5 μg/cm^2^ did not affect the mitochondrial activity assessed using Alamar blue (Figure 3). A significant increase in LDH release was, however, observed in the highest ALI exposure concentration (5 μg/cm^2^) (Figure 4). In the submerged exposures, no cytotoxicity was observed following co-culture exposure to CeO_2_ NPs at cell doses between 1–22 μg/cm^2^ in either of the two assays (Alamar blue and LDH) (Figure 3 and Figure 4). A clear effect was observed for the positive control (Triton X).

### 3.4. Inflammatory Potential in Co-Cultures

**Figure 5:**
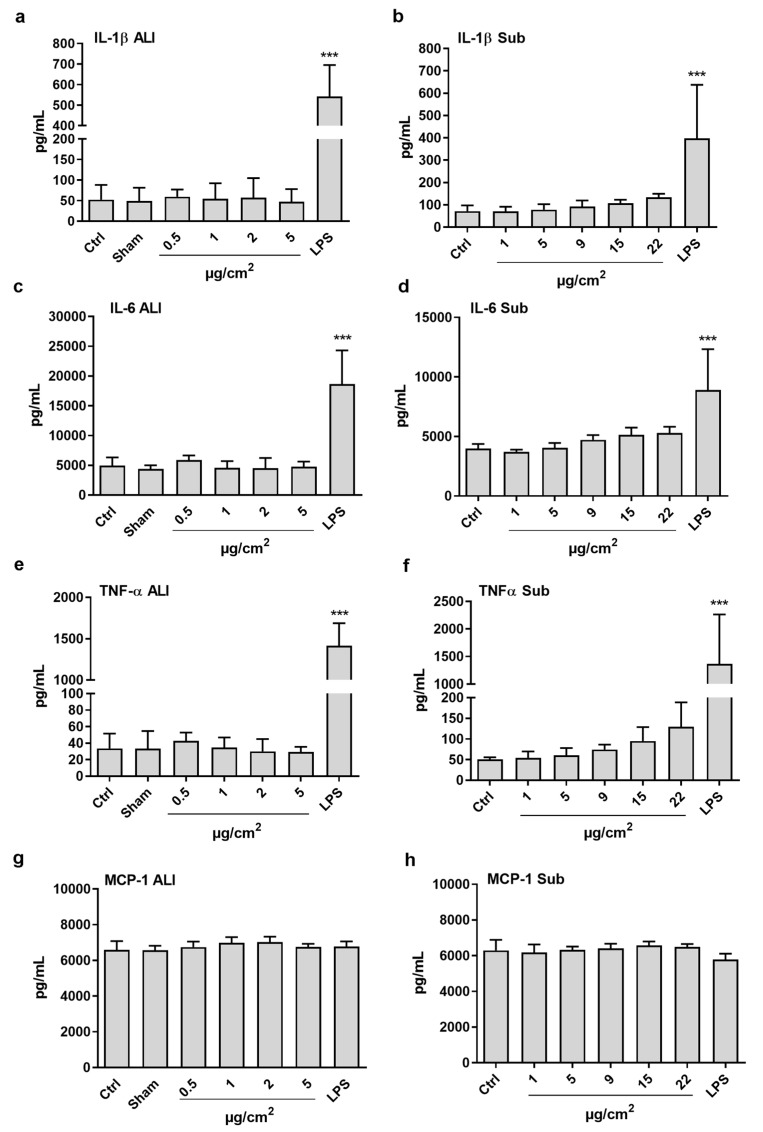
Quantification of cytokine release (IL-1β, IL-6, TNFα, MCP-1) after 24 h exposure to CeO_2_ NPs in air-liquid interface (ALI) (**a**,**c**,**e**,**g**) or in submerged (**b**,**d**,**f**,**h**) co-culture of A549 and THP-1 cells. LPS (100 ng/mL) treatment was used as positive control. The bars represent mean ± SD. Significant results, as compared to the Sham (clean air) for ALI and control (Ctrl) for submerged, are marked with asterisks (*** for *p* < 0.001).3.5. Cytotoxicity and IL-1β Release in THP-1 Cells (Monocultures in Plates)

CeO_2_ NP exposure did not induce a statistically significant increase in the release of the tested inflammatory cytokines (IL-1β, IL-6, TNFα, MCP-1) in the ALI or the submerged co-cultures at the tested concentrations (Figure 5). Only a slight increase of TNFα was observed in the highest CeO_2_ concentration of the submerged exposures (22 μg/cm^2^), although this increase was not statistically significant. The positive control (LPS) caused a substantial release of IL-1β, IL-6, and TNFα, but not of MCP-1.

**Figure 6:**
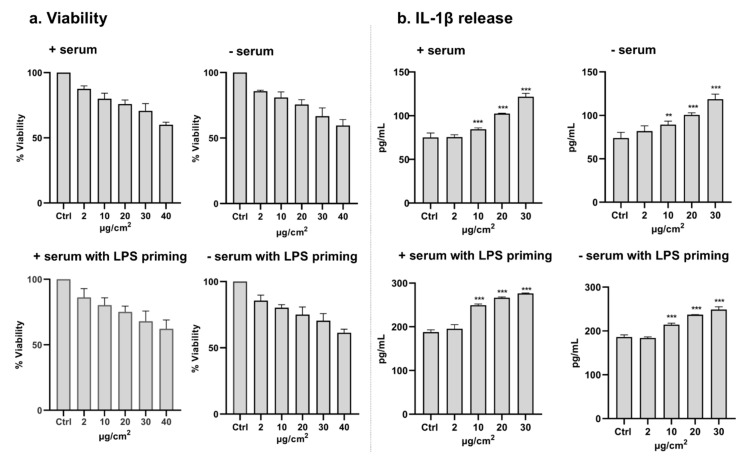
Cell viability (**a**) and IL-1β release (**b**) after 24 h exposure of THP-1 (with or without priming using LPS, 100 ng/mL for 4 h) to CeO_2_ NPs (nominal dose) in submerged conditions in media with or without serum. For viability, the bars represent mean ± SD from three independent experiments. All conditions caused a dose-dependent decrease in cell viability. For IL-1β release, the bars represent mean ± SD from 4 wells in two different experiments. Significant results, as compared to control (Ctrl) are marked with asterisks (**for *p* < 0.001 and *** for *p* < 0.001).

To further explore the inflammatory potential of CeO_2_ NP at different exposure conditions, the role of serum and LPS-priming for IL-1β release was further tested in macrophage-like THP-1 cells in monocultures. All exposure conditions led to some cytotoxicity, with no clear differences dependent on serum and LPS priming (Figure 6). Furthermore, a significant increase in IL-1β release was observed at all four conditions tested (Figure 6), starting from the dose of 10 μg/cm^2^.

## 4. Discussion

The main aim of this study was to compare exposure in ALI and submerged conditions for detecting cytotoxicity and inflammatory effects, following exposure of a co-culture to CeO_2_ NPs. An additional aim was to further explore the role of serum and priming for IL-1β release, following exposure of THP-1 cells. Our results show no clear effect on inflammatory markers in the co-culture model neither at ALI nor submerged exposure. Thus, no clear conclusions can be drawn regarding the ability of the different exposure systems to detect an inflammatory response in the co-culture. However, when differentiated THP-1 cells were exposed (submerged), an increased release of IL-1β was observed for all conditions independently of serum and LPS-priming.

For the ALI exposure, we used the PreciseInhale system in combination with the Xpose*ALI* cell exposure unit. These systems have previously been used to generate dry aerosols and expose cultured cells to palladium (Pd) NPs [20], and diesel exhaust particles [21], as well as carbonaceous model (Printex 90) NPs [22]. A general difficulty in comparing different exposure set-ups is the dose comparison [23]. To allow for direct comparisons between these two exposure methods, the cells were cultured at similar conditions (on inserts), and cell doses were analyzed quantitatively (using ICP-MS) and expressed as µg/cm^2^ for both ALI and submerged exposures. It should be noted that this “cell dose” is not a measure of only the intracellular particles, but a combination of particles on the cells and in the cells. Furthermore, the dose rate was not analyzed, and is likely to be different between the two exposure conditions. At submerged exposure, an alternative approach is to estimate the delivered dose by modeling using e.g., the in vitro sedimentation, diffusion and dosimetry (ISDD) model [24]. This approach has previously been used to compare doses in ALI and submerged conditions [17,25]. In our ALI system, we were able to successfully generate and deposit CeO_2_ NPs from dry powder onto the co-cultures in doses between 0.5–5 µg/cm^2^. Although experiments using ALI systems in general are quite challenging and more time consuming compared to submerged exposures, some advantages with using the PreciseInhale/Xpose*ALI* platform were noted when compared to our previous experiences [8,26]. These include the low amount of powder (mg) needed for the whole study, as well as the relatively short time needed for deposition in the Xpose*ALI* unit (approx. 5–20 min, depending on dose). However, with the inserts and co-culture system used, we noted a flux of medium from the lower into the upper compartment with time. Thus, at the end of the 24 h incubation time, several tens of microliters were noted in the upper compartment.

Other studies have also compared ALI and submerged conditions with diverging results [17,18,23,25]. Our study can be directly compared to the study performed by Loret et al. (2016), which used a very similar approach for toxicity testing the same CeO_2_ NPs (NM-212) [17]. In line with our study, that study showed no increase in TNFα in the ALI exposure, however, a significant increase in IL-1β and IL-6 for the highest dose (3 µg/cm^2^) was observed. Furthermore, no or very minor effects were observed for submerged exposure in inserts (max 10 µg/cm^2^) or submerged exposure in plates (max 20 µg/cm^2^). In a follow-up study, the same team performed in vivo experiments and investigated whether the co-culture at different exposure conditions could predict the in vivo data [27]. Overall, inflammatory effects (IL-1β, IL-6, TNFα in bronchoalveolar lavage) were observed at lower doses in the in vivo experiments, and the data from the ALI exposure was most predictive. The authors speculated that the use of serum in the in vitro experiments may affect the results, and that better correlations may be observed at serum-free conditions.

Inspired by the speculations of Loret et al. [27], we performed experiments on THP-1 (monocultures, submerged) in order to study the role of serum and to investigate whether LPS-priming of the macrophages would lead to a more sensitive assay. First, we noted a dose-dependent decrease in viability, and a relatively small, but significant, increase in IL-1β secretion at all conditions, starting from the nominal dose of 10 µg/cm^2^ (Figure 6). Thus, the monocultures of THP-1 cells (exposed in plates) appeared to be more sensitive compared to the co-cultures of A549 and THP-1 cells (exposed in inserts). One possible explanation (that we did not investigate) may be a higher uptake at such conditions. One other hypothesis for the effect observed in the monoculture is the higher number of macrophages per surface. In the co-culture, 42,000 cells were seeded in the 0.9 cm^2^ transwell (47,000/cm^2^), whereas in the plates, 40,000 were seeded in 0.3 cm^2^ (133,000/cm^2^). The inflammatory effect was observed even though the CeO_2_ NPs were highly agglomerated at serum-free conditions. Apart from the different number of macrophages per surface, as well as the dispersion protocols used, there are some other discrepancies that make a direct comparison difficult, including different amounts of PMA used for differentiation of THP-1 cells (lower PMA in the mono-culture). The same dispersion protocol and PMA concentration used in our co-culture were, however, used in a study by Battacharya et al. (2017) [28], also showing IL-1β release after THP-1 cell exposure to the same CeO_2_ NPs (NM-212). Different sensitivities of dissimilar macrophage-like cells were highlighted in a study by Cho et al. (2013) [29]. THP-1 cells (differentiated with PMA) exposed to CeO_2_ NPs led to an evident release of IL-1β, which appeared to be dependent on phagocytosis, since a reduced effect was observed when phagocytosis was inhibited using cytochalasin D. In contrast, no increase in IL-1β release was observed when exposing monocytic (non-differentiated) THP-1 cells, primary cultured alveolar macrophages, or differentiated PBMC (peripheral blood mononuclear cells). Taken together, our study and other studies suggest that CeO_2_ NPs cause release of IL-1β in THP-1 cells, findings in line with observations in vivo.

The only effect observed in the co-culture was an increase in LDH release in the highest dose tested. Since we only noted this effect in the ALI exposure, it may suggest that cells cultured at such conditions are more sensitive compared to cultures at submerged conditions. Clear conclusions are, however, difficult to draw, since this only was observed in one dose. An increased LDH release, but a lack of inflammatory response, was observed in a study on rats exposed to CeO_2_ NPs [30]. In line with the inflammatory effects observed in macrophage-like cells in our study, Wiemann and colleagues reported inflammatory effects (TNFα release) following exposure of rat alveolar macrophages (NR8383 cells) to the same CeO_2_ material (NM-212) in doses of 22 µg/mL, as well as LDH release at higher doses (>90 µg/mL) [31]. In vitro-in vivo comparisons and correlations are important to perform in order to understand to what extent in vivo studies can be replaced by in vitro studies. One interesting question is the dose comparison. In a study on iron oxide NPs, Teeguarden and co-workers concluded that when considering target tissue dosimetry, especially with a focus on macrophages, a good conformity between target cell doses triggering inflammatory processes in vitro (8–35 pg/cell) and in vivo (1–100 pg/cell) was observed [32].

## 5. Conclusions

This study demonstrates the applicability of the PreciseInhale system for the generation of dry aerosols from particle powders, and the exposure of cell cultures to these aerosols in the Xpose*ALI* unit. This approach allows for thorough comparisons between ALI and submerged exposure conditions, which are important in order to better understand the possible differences in toxicological responses between these two exposure methodologies. The tested CeO_2_ NPs (NM-212) showed low cytotoxicity and inflammatory potential, following the exposure of A549 and THP-1 co-cultures in both exposure systems. Exposure to a monoculture of THP-1 cells led to a clear IL-1β release, suggesting this model to be more sensitive compared to the co-culture at submerged conditions.

## Figures and Tables

**Table 1:** The deposited mass of CeO_2_ NPs (mean of 4 transwell inserts ± SD) at ALI and submerged (sub.) conditions, determined by means of ICP-MS after 24 h. Added mass refers to the weighed amount of CeO_2_.

Deposited Mass ALI (μg/cm^2^)	Deposited Mass sub. (μg/cm^2^)	Added Mass sub. (μg/cm^2^)
0	0	0
0.5 ± 0.05	1 ± 0.4	2
1 ± 0.2	5 ± 0.6	10
2 ± 0.6	9 ± 1.5	20
5 ± 0.7	15 ± 1.1	30
**-**	22 ± 1.9	40

**Table 2:** CeO_2_ size and sedimentation in cell medium. The characterization of the CeO_2_ NPs was assessed using photon cross correlation spectroscopy (PCCS), following sonication of the stock suspensions using different techniques followed by dilution to 20 μg/mL.

	Size (nm)		Scattered Light Intensity (Kcounts/s)	
	0 h	24 h	0 h	24 h
Probe sonication (MilliQ + albumin)	236 ± 11	235 ± 17	2150 ± 56	868 ± 241
Bath sonication (+ serum)	865 ± 238	396 ± 47	1396 ± 186	265 ± 122
Bath sonication (- serum)	3279 ± 177	-	1392 ± 35	8 ± 8

- no agglomerates in solution.
